# Preparation of Carbon-Alumina (C/Al_2_O_3_) aerogel nanocomposite for benzene adsorption from flow gas in fixed bed reactor

**DOI:** 10.1016/j.mex.2019.10.019

**Published:** 2019-10-18

**Authors:** Ayoob Rastegar, Mahdi Farzadkia, Ahmad Jonoidi Jafari, Ali Esrafili

**Affiliations:** a Department of Environmental Health Engineering, School of Public Health, Sabzevar University of Medical Sciences, Sabzevar, Iran; b Department of Environmental Health Engineering, School of Public Health, Iran University of Medical Sciences, Tehran, Iran

**Keywords:** Benzene removal from flow gas by aerogel nanocomposite Carbon-Alumina (C/Al_2_O_3_), Benzene adsorption, Aerogel, Carbon-Alumina, Nanocomposite

## Abstract

Benzene is one of the hazardous environmental pollutants which have harmful effects on human, animal and environmental health. the goal of this study was to synthesize a carbon/alumina aerogel with large surface area, which is easily recoverable and can be used as an adsorbent for benzene removal from the polluted air flow. The composite aerogel was dried at ambient temperature for 48 h and at 120 °C for 48 h in a sequential way. The characteristic properties of the composite aerogel were analyzed using Brunauer-Emmett-Teller (BET), Scanning Electron Microscopy (SEM), Barrett-Joyner-Halenda (BJH) method and energy-dispersive X-Ray (EDX) techniques. The findings of this study showed that, with increasing of the inlet flow rate from 0.2 l/min to 0.5 and 0.8 l/min, the breakthrough time decreased from 13 h to 7 and 3 h, respectively., Besides, the amount of adsorption capacity on aerogel nanocomposite decreased from 126.73 mg g^−1^ to 84.5 and 38 mgg^−1^ with increasing the inlet benzene concentration from 100 ppmv to 200 and 300 ppmv, respectively. In addition, the a carbon/alumina aerogel can be exploited for removing other pollutants from air and water.

Recently, the adsorption process has attracted many research attentions for Benzene removal from different applications due to some inherent characteristics such as low-cost and high feasibility.

• The results indicated that the synthesis of the composite aerogel have been successfully performed.
• The composite aerogel was synthesized by combining Novollac with alumina, which had high surface area and suitable benzene adsorption ability.

## Graphical abstract

**Figure:**
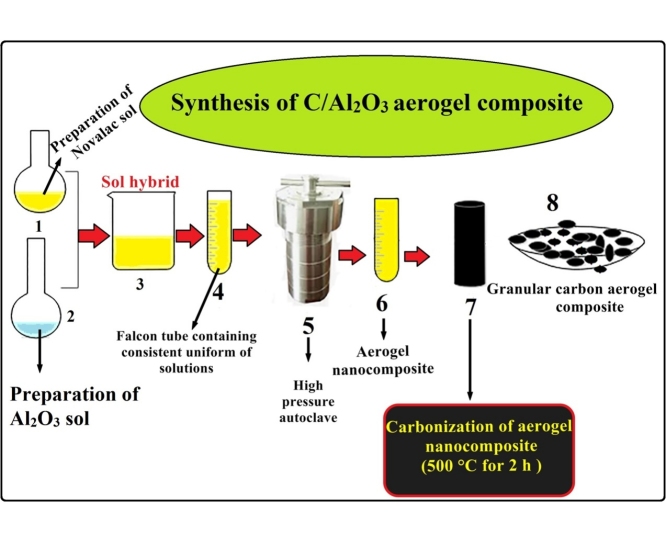

**Specification Table**

**Table:** 

Subject area:	Environmental Science
More specific subject area:	Adsorption
Method name:	Benzene removal from flow gas by aerogel nanocomposite Carbon-Alumina (C/Al_2_O_3_)
Name and reference of original method:	Synthesis of a novel porous material made up of carbon/alumina composite aerogels monoliths with high compressive strength, Microporous and mesoporous materials, 172: 182-189 (2013)
Trial registration:	Not applicable
Ethics:	Not applicable
Resource availability:	The data are available with this article.

## Method details

### Background

Volatile Organic Compounds (VOCs) with high toxicity and carcinogenetic characteristics are well known as compounds with great of concern among different air pollutants [1]. The VOCs are emitted from various industrial activities such as automobile exhausts, petrol and gasoline evaporation, solvent paints, and organic solvents [2,3]. Amongs the different compounds of VOCs group; Benzene is classified as a carcinogenic compound by the international agency for research on cancer (IARC) [4]. Long-term to Benzene compounds leads to a range of severe adverse health effects such as hematologic cancers, destructive effects on the immune system, and aplastic anemia [5]. Therefore, on-site emission control and removal of benzene before release to the atmosphere are so importantin view point of health. The adsorption process is an acceptable method for removal of VOCs from polluted air. Several adsorbents such as bagasse ash [6], zeolite [7], and activated carbon have been applied as adsorbents for the removal of VOCs from the air [2,[8], [9], [10]]. However, the efficiency of these adsorbents is nearly low due to pore blocking, low specific surface area, and decrease in adsorption capacity after regeneration [11]. Aerogels with high specific surface areas, low density, high selectivity and high porosities show promise for removal VOCs componets from polluted air flow [11]. Also, the adsorption capacity of aerogels does not change even after a long period and using for many adsorption-desorption cycles [12]. However, it is also responsible for the relatively poor mechanical behavior of this material which is typically expressed as fragility and brittleness [13]. Therefore, enhancing the mechanical properties of aerogel is essential to broaden the range of its application. For this purpose, in recent years, various materials, including polymers organic, carbon nanotube, and activated carbon have been used to enhance the mechanical strength of inorganic aerogel [[14], [15], [16], [17]]. Resorcinol-formaldehyde (RF) can promote the mechanical structure of aerogel; it is converted to carbon through the pyrolysis process at the temperature range of 750–1500 °C. In this sense, several composite aerogels, including Al^1^ -RF^2^, Nb_2_O_5_^3^ -RF, and TiO_2_-RF, SiO_2_-RF have been prepared and in addition, the enhanced catalytic and adsorption efficiency have been reported [[18], [19], [20], [21]]. Studies indicated that the addition of RF to alumina aerogel structure increased the specific surface area and total pore volume [13,22]. However, these compounds are toxic, dangerous and costly.

To overcome thesedrawbacks, a group of researchers reported that the Novolac is a low-price commercial resin with high char-yield efficiency and pyrolysis of Novolac-containing aerogels leads to the formation of carbon aerogels with significant properties [23]. As regards, the use of Novalac polymer has not been investigated to increase mechanical strength alumina aerogel. In the present research, the Novolac/alumina aerogel (N/Al_2_O_3_) was prepared using the sol-gel technique and ambient drying. Finally, the structures and properties of carbon–alumina aerogel as adsorbents were characterized to fill the knowledge gap. Therefore, the present study aimed to evaluate the removal efficiency of composite aerogel (C/Al_2_O_3_)of removal of benzene from contaminated air.

### Chemical reagents

Aluminum chloride hexahydrate (AlCl_36_H_2_O) and Novolac resin (made in Iran with 9% wt of hexamethylenetetramine (HMTA)) were applied as an alumina source and organic phase precursor, respectively. Other chemical materials such as 2-propanol, deionizer water, and propylene oxide (PO) were used as solvent, hydrolysis agent and crosslinking, respectively. All the chemical materials and solutions were of analytical grade and used without further purification.

### Preparation of composite aerogel (C/Al_2_O_3_)

**Fig. 1:**
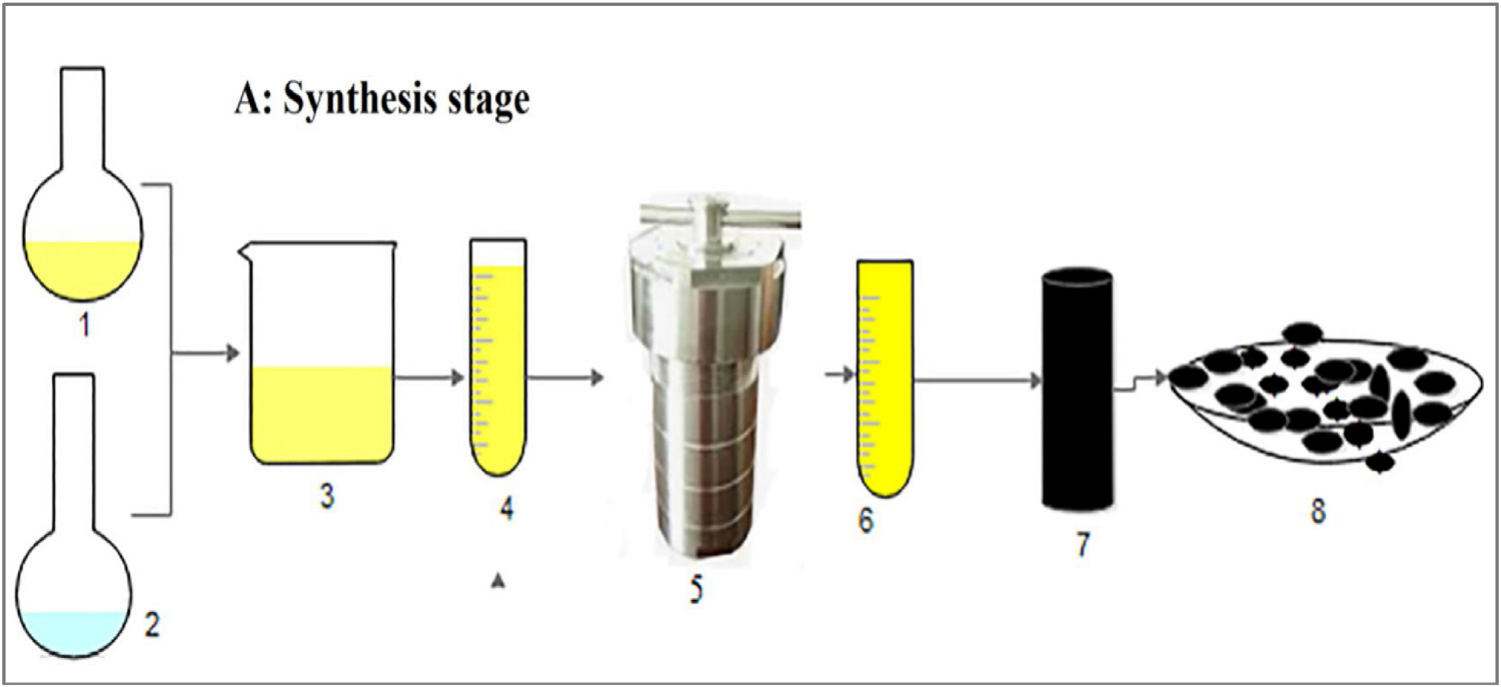
The schematic diagram of synthesis aerogel composite: (**1**) Novalac sol, (**2**) Al_2_O_3_ sol, (**3**) Sol hybrid, (**4**) Falcon, (**5**) High-pressure autoclave, (**6**) Aerogel composite, (**7**) Carbonization of aerogel nanocomposite, (**8**) Granular carbon aerogel composite.

A schematic diagram of the synthesis composite aerogel (C/Al_2_O_3_) is illustrated in Fig. 1.

The sol-gel method was applied to synthesis of C/Al_2_O_3_ according to the following steps (Fig. 1):

1 Preparation of sol A (Novalac sol): 15 g Novolac resin dissolved in 85 ml 2-propanol and using ultrasonic at a frequency of 20 kHz for 30 min at room temperature.
2 Preparation of sol B (Al_2_O_3_ sol): At the same time, Al_2_O_3_ sol was obtained by mixing 24.1 g aluminum chloride hexahydrate, 81 g of deionized water, 41.4 g of absolute ethyl alcohol, and 58 g of propylene oxide.
3 Both sols (A and B), before converting to gels, were mixed thoroughly and exposed to ultrasound for 30 min. It should be noted that the molar ratio of Novolac to Al_2_O_3_ was fixed with a ratio of 4:1.
4 The consistent uniform of solutions was poured into a falcon tube with the interlayer distance of 3 cm.
5 The solution was placed inside a high-pressure autoclave containing a low amount of 2-propanol and treated at 105 °C for five hours and then cooled down to room temperature.
6 The resulting gel was dried at ambient temperature for 48 h and subsequently dessicated at 120 °C for 48 h.
7 Subsequently, the prepared aerogel nanocomposite was carbonized at 500 °C for two h under nitrogen gas flow rate of 10 ml/min. In this stage, composite aerogel preparation was grained and sieved with 20–40 mesh.
8 Granulation of N/Al_2_O_3_: The N/Al_2_O_3_ composite aerogel was converted to C/Al_2_O_3_ at 1000 °C for 3 h under nitrogen gas flow with a rate of 10 ml/min [13,23].

### Characterization

The C/Al_2_O_3_ composite aerogel was placed in cylinder cast, and the specific area of the aerogel (C/Al_2_O_3_) was characterized through measurement of adsorption nitrogen gas at −196 °C by Belsorb mini II device, and the elements existing in the synthesized sample were determined with Energy-dispersive X-ray spectroscopy (EDX) analysis (ARL X_0_ TRA diffract meter (Rigaku)) with Cu-Ka radiation (30 kV, 30 mA). The morphology of the structure sample was characterized using an LEO-1530VP field emission scanning electron microscope (SEM) operating at 10 keV. Joyner-Halenda (BJH) method was applied to measure the pore size distribution (PSD) of aerogel composite.

#### Aerogel characterization

Table 1 presents the information on the total specific pore volume, surface area, average pore diameter, external surface area of the aerogel composite. The C/Al_2_O_3_ has a high specific surface area (762.97 m^2^/g) with a total pore volume of 0.59 cm^3^/g and 3.29 nm pores in diameter.

**Table 1:** Characteristics of synthesized sample.

Samples	S_BET_ (m^2^/g)	S _Meso_ (m^2^/g)	S _micro_ (m^2^/g)	S _exit_ (m^2^/g)	Pore V_total_ (cm^3^/g)	Diameter pore (nm)	Monolayer volume (cm^3^ g^−^
C/Al_2_O_3_	762.97	904.67	88	43.10	0.59	3.09	207.85

**Fig. 2:**
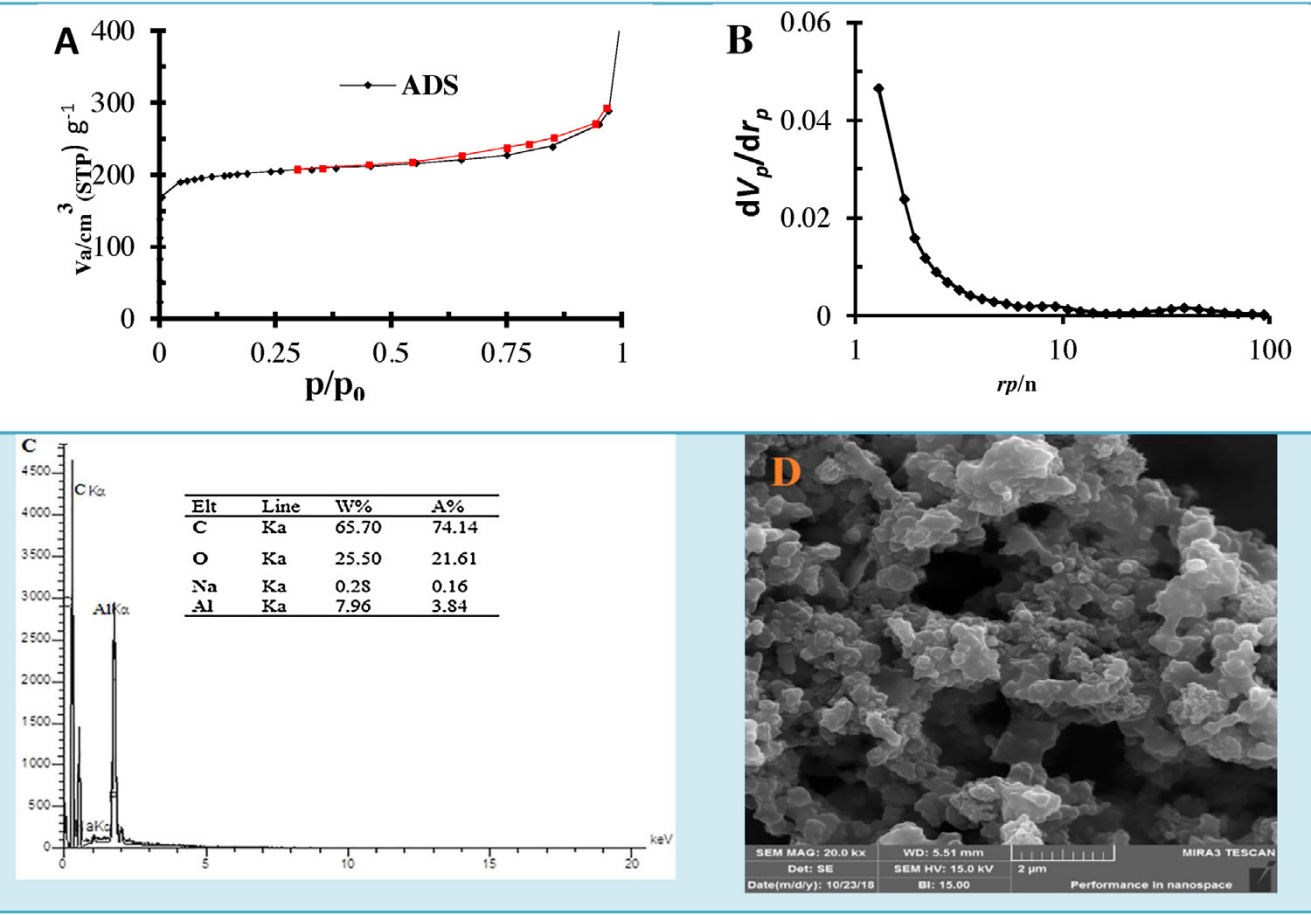
Elemental analysis and morphology of C/Al_2_O_3_: **(A)** Nitrogen adsorption-desorption isotherms, **(B)** Pore size distribution, **(C)** EDAX, (**D**) SEM.

Fig. 2(A) shows the adsorption/desorption isotherms of the composite aerogel (C/Al_2_O_3_). The results indicated that the adsorption-desorption isotherm is fitted with type IV with H1-type hysteresis loop, indicating a porous structure with cylindrical pores. Fig. 2(B) shows the pore size distributions of the composite aerogel (C/Al_2_O_3_). The pore size distribution is estimated using the Barrett-Joyner-Halendar method. As observed in this figure, single pores with the maximum pore size in the range of 2–12.17 nm. Therefore, according to the IUPAC pore size are mesoporous. Also, Fig. 2(C) shows the presence of chemical elements (Al, C, and O) in the building of the prepared aerogel composite. These results indicate the existence of C (65.7%), O (25.5%) and Al (7.9%) elements in the composites. The FESEM analysis (Fig. 2(D)) shows that the spherical solids are distributed uniformly over the surface. It is suggested that the existence of formation cylindrical pores in the structure of aerogel composite, explaining of suitable porosity for sorption of contaminants.

### Experiments of adsorption in dynamic conditions

**Fig. 3:**
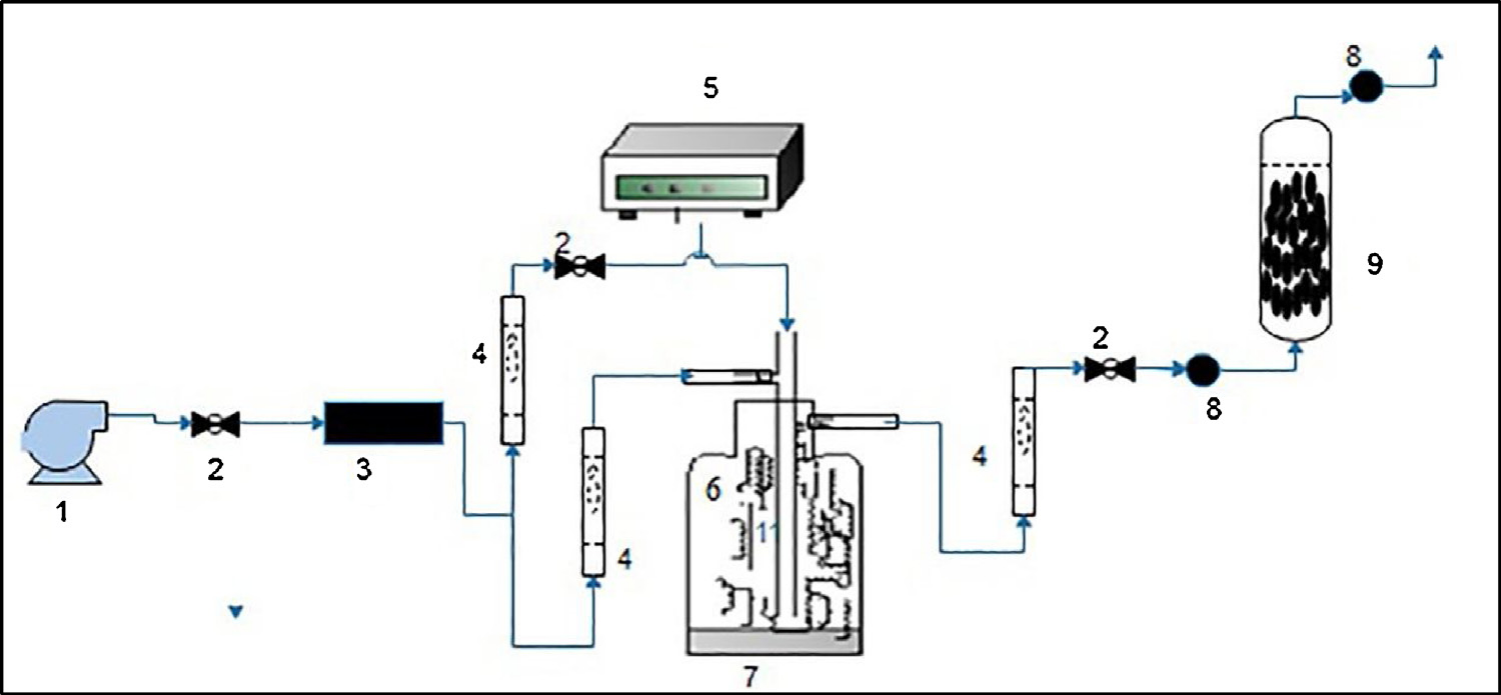
Schematic of the experimental reactor: (1) Air pump, (2) Needle valve, (3) Activated carbon adsorbents, (4) Rotameter, (5) Needle pump, (6), Benzene vapors (7) mixing chamber, (8) Sampling port.

Fig. 3 indicates the schematic diagram of the experimental set-up used in the present study.

Fig. 3 indicates the schematic diagram of experimental set-up used in the present study. In this study, an air pump was employed to provide air flow rate and remove impurities transformed from an activated carbon column. The pure air was transported into mixing chamber to which liquid benzene was injected by a syringe pump (2 ml h^−1^). The air flow rate of entering to mixing chamber was adjusted at 7 l min^−1^ to obtain the air containing benzene with a concentration of 100 ppm. All experiments runs were performed in a fixed-bed continuous-flow reactor with internal diameter of 1.5 cm and a height of 30 cm. A glass gauge filter was installed in the bottom of the column to make sure of uniform distribution of the flow air into reactor.

Then, 1 g of fine particles of composite aerogel was loaded inside the column in each step of experiments to determine the adsorption capacity of aerogel composite. The sampling from designated locations was performed by a gas tight syringe for determination of the concentrations of benzene. Then, the amount of 100 μL was injected into the gas chromatography (Varian CP-3800), equipped with a detector (FID) and column (0.25 m inner diameter and 15 m length), to determine the concentration levels of benzene. The temperature program of the column was set from 50 to 150 °C with an increasing ramp of 2 °C min. Injection temperature of 240 and the injection volume was set to split with a ratio of 100:5. In addition, a temperature of 250 °C was set for the detector. At the end, the following equation (Eq. (1)) was applied to compute the quantity of adsorption capacity in the breakthrough point:

(1) $$\text{BC}={(\text{C}}_{\text{in}}\times \;{\text{T}}_{\text{bk }}\times \text{Q})/{\text{M}}_{\text{adsorbent}}$$

Where,

BC: Adsorption capacity of benzene on a gram of adsorbent when it reaches a breakpoint (mg/g)

C_in_: Input concentration of benzene (mg/m^3^)

T_bk_: breakthrough time (h)

Q: Air flow rate (m^3^)

M: The amount of applied adsorbent (g)

### Adsorption process study

#### The effect of flow rate on benzene removal

**Fig. 4:**
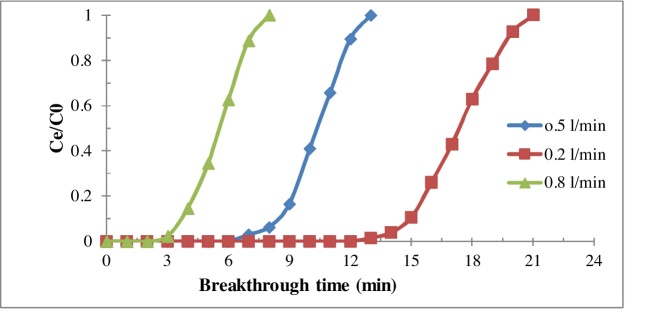
Effect of flow rate on the efficiency of aerogel in the benzene removal (Concentration of inlet benzene: 300 ppm; EBCT: 2.1 s; Absorbent dosage: 2 g).

The breakthrough time of composite aerogel for removal of benzene was studied at different flow rate. The findings in Fig. 4 indicate that, with increasing flow rate from 0.2 l/min to 0.5 and 0.8 l/min, the breakthrough time of the benzene onto composite aerogel diminished from 13 h to 7 and 3 h, respectively. The decrease of breakthrough time with the increase of flow rate can be attributed to low expose time between benzene molecules with active sites on the surface of the composite aerogel [24]. Additionally, shorter breakthrough time can be due to transporting more mass of benzene inside the bed. Hence, the active sites of the composite aerogel quickly are saturated and breakthrough time occurs in relatively slower time. According to similar studies, the breakthrough time with the increase of flow rate was decreased [25,26]. These results are in agreement with the other studies results [24].

#### Effect of benzene concentration

**Fig. 5:**
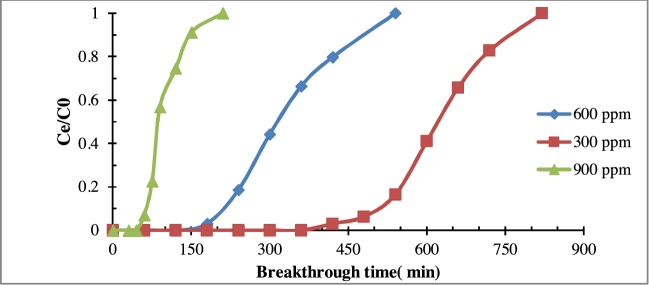
Effect of inlet benzene concentration on the efficiency of aerogel (Operational conditions: EBCT = 2 s; Flow rate = 0.5 Lm^−1^, Adsorbent dosage=2 g).

The effect of benzene concentrations on the breakthrough time were studied at different inlet concentrations. The Fig. 5 indicates that the breakthrough time decreased from 420 min to 180 and 60 min with increasing of the Benzene concentration from 300 ppmv to 600 and 900 ppmv, respectively. Besides, Eq. (1) was employed to calculate removal capacity in the breakthrough time by C/Al_2_O_3_ aerogel composite. The findings also showed that the amount of adsorption capacity on C/Al_2_O_3_ decreased from 126.73, 84.5 to 38 mg g^−1^ by increasing the inlet Benzene concentration. It can be explained by the fact that more mass was transferred to the adsorbent surfaces [25,27,28].

#### Reusability aerogel composite

**Fig. 6:**
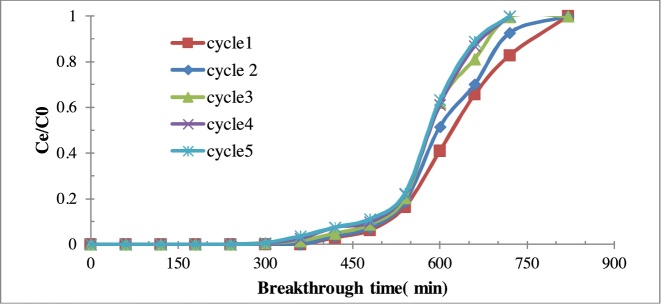
Breakthrough time for benzene in five cycles of the adsorption-desorption experiment by C/Al_2_O_3_ composite aerogel (Concentration of inlet benzene: 300 ppm; Adsorbent dosage: 2 g; total flow rate: 0–5 l/min).

To the adsorption properties of aerogel composite repeated adsorpion/desorption cycles of benzene on aerogel composite was performed. The regeneration of composite aerogel performed by extraction of adsorbed benzene and drying of sample at 250 °C under vacuum conditions. Fig. 6 shows the breakthrough time curves of C/Al2O3for benzene vapors. The findings indicate that the differences between breakthrough time in the first and five series are negligible. It can be related to the stability of pours due to the entry and exist of benzene molecules into it. Also, it can be concluded that adsorption of benzene molecules on the aerogel composite was poor and adsorbents molecules can be separated by thermal purification [29]. Hong Sui has reported that the breakthrough time for silica gel and activated carbon commercial were different when the recycling time is increased from the first cycle to five one This difference is due to the remain of toluene molecules on the adsorbent after the desorption step [29,30].

## Footnotes

1 Aluminium.

2 Resosinol-formaldehyde.

3 Niobium pentoxide.
